# Determination of aortic stiffness using 4D flow cardiovascular magnetic resonance - a population-based study

**DOI:** 10.1186/s12968-018-0461-z

**Published:** 2018-06-21

**Authors:** Andreas Harloff, Hanieh Mirzaee, Thomas Lodemann, Paul Hagenlocher, Thomas Wehrum, Judith Stuplich, Anja Hennemuth, Jürgen Hennig, Sebastian Grundmann, Werner Vach

**Affiliations:** 1 Department of Neurology, Medical Center – University of Freiburg, 79106 Freiburg, Germany; 2 Faculty of Medicine, University of Freiburg, Freiburg im Breisgau, Germany; 3 Fraunhofer MEVIS, Bremen, Germany; 4 Department of Cardiology and Angiology I, Heart Center Freiburg University, University of Freiburg, Freiburg im Breisgau, Germany; 5 Charité-Universitätsmedizin Berlin, Berlin, Germany; 6 Department of Diagnostic Radiology – MR Physics, Medical Center - University of Freiburg, Freiburg im Breisgau, Germany; 7 Institute for Medical Biometry and Statistics, University of Freiburg, Freiburg im Breisgau, Germany; 8 Department of Orthopedics & Traumatology, University Hospital Basel, Basel, Switzerland

**Keywords:** Aorta, Stiffness, Population-based study, 4D flow MRI, Pulse wave velocity

## Abstract

**Background:**

Increased aortic stiffness is an independent predictor of cardiovascular disease. Optimal measurement is highly beneficial for the detection of atherosclerosis and the management of patients at risk. Thus, it was our purpose to selectively measure aortic stiffness using a novel imaging method and to provide reference values from a population-based study.

**Methods:**

One hundred twenty six inhabitants of Freiburg, Germany, between 20 and 80 years prospectively underwent 3 Tesla cardiovascular magnetic resonance (CMR) of the thoracic aorta. 4D flow CMR (spatial/temporal resolution 2mm^3^/20ms) was executed to calculate aortic pulse wave velocity (PWV) in m/s using dedicated software. In addition, we calculated distensibility coefficients (DC) using 2D CINE CMR imaging of the ascending (AAo) and descending aorta (DAo). Segmental aortic diameter and thickness of aortic plaques were determined by 3D T1 weighted CMR (spatial resolution 1mm^3^).

**Results:**

PWV increased from 4.93 ± 0.54 m/s in 20–30 year-old to 8.06 ± 1.03 m/s in 70–80 year-old subjects. PWV was significantly lower in women compared to men (*p* < 0.0001). Increased blood pressure (systolic *r* = 0.36, *p* < 0.0001; diastolic *r* = 0.33, *p* = 0.0001; mean arterial pressure *r* = 0.37, *p* < 0.0001) correlated with PWV after adjustment for age and gender. Finally, PWV increased with increasing diameter of the aorta (ascending aorta *r* = 0.20, *p* = 0.026; aortic arch *r* = 0.24, *p* = 0.009; descending aorta *r* = 0.26, *p* = 0.004). Correlation of PWV and DC of the AAo and DAo or the mean of both was high (*r* = 0.69, *r* = 0.68, *r* = 0.73; *p* < 0.001).

**Conclusions:**

4D flow CMR was successfully applied to calculate aortic PWV and thus aortic stiffness. Findings showed a high correlation with distensibility coefficients representing local compliance of the aorta. Our novel method and reference data for PWV may provide a reliable biomarker for the identification of patients with underlying cardiovascular disease and optimal guidance of future treatment in studies or clinical routine.

## Background

Atherosclerosis develops over a time period of several decades, but is usually not perceived by individuals until symptoms occur. During this period, cumulative vessel damage develops and is usually irreversible at the time of first cardiovascular event. Aortic stiffness expressed as aortic pulse wave velocity (PWV) is a strong predictor of future cardiovascular events and all-cause mortality [1]. Measuring PWV offers the chance to identify subjects at risk and to intervene timely through life style modifications and medication. In contrast to classical cardiovascular risk factors, PWV is a more stable parameter that gradually becomes abnormal and represents vascular aging [2]. Consequently, it has been recommended as an independent parameter for individual risk assessment [3, 4].

In the past, it was demonstrated that an increase of aortic stiffness is associated with cardiovascular disease [5]. Similarly, decreased proximal aorta distensibility determined by gradient echo phase-contrast cine cardiovascular magnetic resonance (CMR) could independently predict all-cause mortality and severe cardiovascular events including stroke [6]. In most trials, aortic stiffness was assessed by measuring carotid-femoral PWV with tonometry. This technique, however, is hampered by the inaccurate measurement of the distance between the carotid and femoral measurement points and by the inclusion of the stiffness of these vessels. Catheter measurement of aortic PWV is the reference method for assessing aortic stiffness but being invasive and thus not suited for trials [7]. CMR, using multiple 2D slices of the aorta is a non-invasive alternative [8]. However, it is based on only few measurement points and not applicable if the aorta is elongated [9]. 4D flow CMR was recently used for full volumetric assessment of aortic PWV in healthy subjects and acute stroke patients. It showed high accuracy even in patients with complex aortic geometries [10–12]. Therefore, this technique is ideally suited for measuring aortic stiffness. However, normal data of selected aortic stiffness from a population-based study using 4D flow CMR is lacking, which would be prerequisite for providing age- and gender-stratified reference values for patients with yet unknown or clinically manifest cardiovascular disease. As a result, individuals with early deterioration of vascular function, progressive subclinical atherosclerosis, and the need of intensified treatment could be reliably identified.

For these reasons, it was the scope of this study to determine normal values of aortic PWV in a general population by means of a novel method using 4D flow CMR.

## Methods

### Study population

We performed a cross-sectional observational study of the population of the city of Freiburg, Germany, based on data obtained from the local residents’ registration office. Twenty subjects per decade (~ 10 females and ~10 males) between 20 and 80 years of age were consecutively and prospectively recruited.

From October 2012–August 2014, 3500 age-stratified and randomly selected residents were contacted by mail and asked to participate in our study. A total of 308 responded and were contacted consecutively by phone and recruited on a first-come, first-served basis. 147 had to be excluded because of CMR contraindications, too many volunteers in one age group or because no suitable date for CMR examination could be arranged. 161 subjects were scheduled for CMR. In 19 of them, CMR could not be completed for technical reasons (failure of the electrocardiogram (ECG)-trigger or data reconstruction), 11 did not appear in the CMR suite on the appointed date, 11 aborted examination early because of claustrophobia, and 5 were not suited due to contraindications that became evident only on site (ferromagnetic implants). In 7 subjects, CMR data could not be analyzed using the analysis software. Because of insufficient response in the group of 20–29 and 30–39 year old males, the study was advertised on the University Hospital Freiburg intranet for men of that age. As a result, 35 subjects responded and the first 18 subjects who contacted the study team were consecutively included. One of them had to be excluded for technical difficulties during the CMR scan, another did not appear on the appointed day, and in a third transthoracic echocardiography (TTE) could not be performed due to migration to another city. Finally, complete CMR and TTE datasets of 126 (111 + 15) subjects were available for analysis.

The local ethics committee approved the study and written informed consent was obtained from all participants.

### Baseline characteristics

Cardiovascular risk factors and demographics were determined by interview on site. Blood pressure was measured at the left upper arm in a supine position after 5 min rest before and after CMR examination. Heart rate was documented every 5 min during blood flow measurements in CMR.

### Transthoracic echocardiography

All participants underwent TTE using a Toshiba Artida system (4.8–2 MHz PST-30BT transducer; Toshiba Medical Systems Corporation, Tokyo, Japan) based on the recommendations and standards of the American Society of Echocardiography [13]. TTE was performed on the same day as CMR except in 22 patients. In those, TTE was delayed due to organizational reasons and performed 35 ± 31 days after CMR. All standard 2D TTE images, standard M-mode and Doppler images were obtained in apical, parasternal long and short axis view and in subcostal view. Left ventricular (LV) systolic and diastolic function, LV ejection fraction (EF), right ventricular (RV) systolic function, wall-motion and valvular function and morphology were assessed. Left atrial volume, LV and RV dimensions, LV wall thickness and diameters of the ascending aorta were calculated. Flow velocity measurements through the tricuspid, pulmonary outflow, mitral, and aortic outflow regions were performed. The inferior vena cava was imaged in subcostal view.

### Measurement of aortic atherosclerosis

All CMR examinations were conducted on a commercial 3 Tesla CMR sysem (TIM Trio, Siemens Healthineers, Erlangen, Germany), using a commercial 12-element body coil. T1 weighted bright-blood (3D gradient echo sequence, echo time/repetition time (TE/TR) = 1.89/152.53 ms, flip-angle = 20°, acceleration = GRAPPA (*R* = 2, 32 ref. lines) with a spatial resolution of 1.1 × 0.9 × 1.1mm^3^ was applied to measure maximum diameter of the ascending (AAo) and descending aorta (DAo) at the level of the pulmonary artery. ECG-trigger and navigator gating were used to minimize motion artifacts [14]. The aortic arch was defined as the segment between the outlet of the brachiocephalic trunk and the left subclavian artery and arch diameter was measured at the summit and between arteries. Maximum plaque thickness was determined manually in each of these three aortic segments using electronic calipers and a routine picture archiving and communication system (pacs) (IMPAX EE, Agfa HealthCare, Bonn, Germany). We dichotomized circumscribed wall thickening as atheroma < 4 mm and ≥ 4 mm. The latter are considered complex plaques and associated with an increased risk of stroke [15].

### Measurement of aortic blood flow

4D flow CMR was applied to receive time-resolved and 3D blood flow information of the thoracic aorta. All experiments used prospective ECG- and navigator-gating to allow free breathing [11]. Parameters of 4D flow CMR were: TE/TR = 2.54/5 ms, flip angle = 7°, temporal resolution = 20 ms, matrix size = 340x255x75, bandwidth = 450 Hz/pixel, spatial resolution = 2.5 × 2.1 × 2.5mm^3^, velocity sensitivity along all three directions = 150 cm/s, and parallel imaging (PEAK-GRAPPA) along the phase encoding direction (y) with an acceleration factor of *R* = 5 (20 reference lines).

### Calculation of pulse wave velocity

**Fig. 1:**
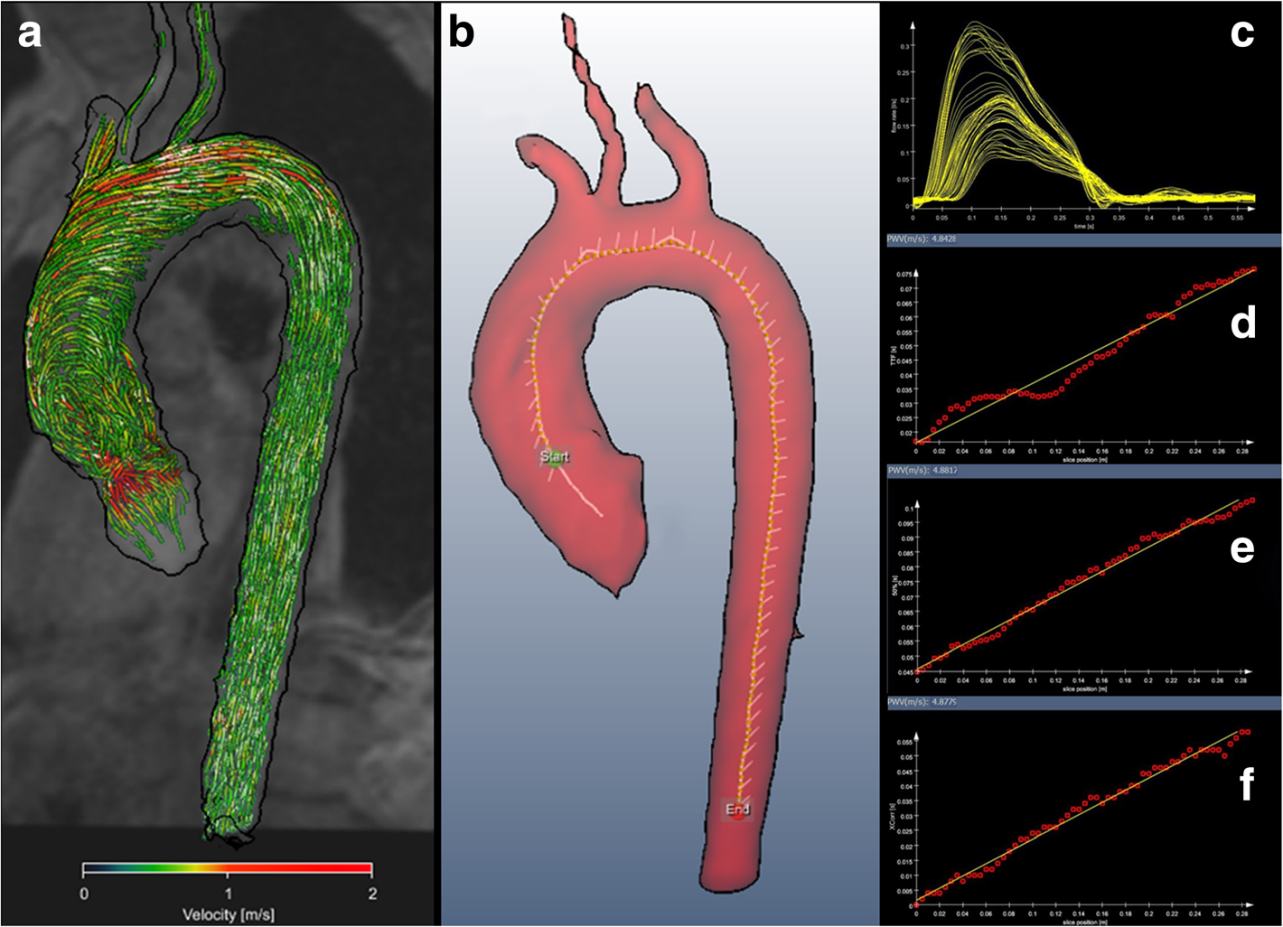
Calculation of pulse wave velocity. 4D flow MRI represents 3D and time-resolved absolute blood flow velocities in the thoracic aorta. In this example, blood flow is visualized using color-coded streamlines (**a**). After vessel segmentation, a centerline and multiple analysis planes (white lines) are automatically positioned perpendicular along the aorta with an inter-plane distance of 5 mm from the starting point (green) to the end point (red) (**b**). Based on time-resolved blood flow velocities PWV is automatically calculated (**c**). Software provides values in m/s based on the time-to-foot (**d**) 50%-rule (**e**) or cross correlation (**f**) method using a linear slope fitted to all single values. PWV in this young subject was 4.84 m/s, 4.88 m/s and 4.88 m/s, respectively

Datasets of 4D flow CMR measurements were analyzed off-line using MEVISFlow software (Fraunhofer MEVIS, Bremen, Germany) [16]. After corrections for eddy-currents and phase-wraps, the aorta was semi-automatically segmented. A centerline was automatically positioned along the entire thoracic aorta starting from the aortic root to the level of the diaphragm. Then, CMR analysis planes were automatically distributed along the centerline after manually setting start and end points within the aortic lumen. They were orientated normal to the aorta with an inter-plane distance of 5 mm. This resulted in 61 ± 7.4 analysis planes depending on individual length of the thoracic aorta (30.0 ± 3.7 cm) (see Fig. 1). A lumen contour surrounding the lumen was defined automatically and adapted to all time points of the cardiac cycle [16, 17].

Individual pulse wave velocity in m/s was calculated based on these planes using the time-to-foot (TTF), 50%-rule (time point where the flow rate is half of the peak flow rate) and cross correlation (XCor) as described previously [11, 12, 17]. Automated computing of PWV using all three algorithms took about 3–5 min when using a 2.7 GHz Intel Core i5 computer with a 12Gb RAM working memory.

For the evaluation of inter-observer agreement of PWV calculation, another reviewer (#2) who was blinded to the results of reviewer #1 and to the characteristics of the study subjects repeated PWV calculations in a subgroup using the same software version. Both observers have more than five years of experience with 4D flow CMR and MevisFlow software.

Randomization of 25 out of 126 subjects was performed using www.randomization.com in order to randomly cover different age groups and sex. After a short instruction by observer #1, observer #2 independently repeated all analysis steps including vessel segmentation, definition of start and end of the centerline, positioning of analysis planes and automated calculation of PWV using the cross-correlation method.

### Calculation of aortic distensibility coefficients

In addition to PWV, we evaluated 2D CINE imaging for the calculation of the distensibility coefficient (DC), a marker of local aortic compliance [18]. 2D CINE imaging was performed in the ascending and descending aorta at the level of the pulmonary artery in each subject in order to identify the individual optimal time delay for the subsequent execution of 3D T1 weighted CMR. It allowed determining the time period with minimal motion of the aorta during the cardiac cycle. We used 2D FLASH CINE with retrospective ECG gating and breath-hold for ≤10s with the following parameters: number of phases = 25; FOV = 340 × 276 mm^2^, slice thickness = 6.0 mm; matrix size = 192 × 154; repetition/echo time = 5.1/2.47 ms; pixel bandwidth = 449 Hz/px; reconstructed voxel size = 1.8 × 1.8x6mm^3^; temporal resolution ~ 40 ms; flip angle = 12°; parallel imaging mode = GRAPPA (acceleration factor = 2, number of reference lines = 30).

**Fig. 2:**
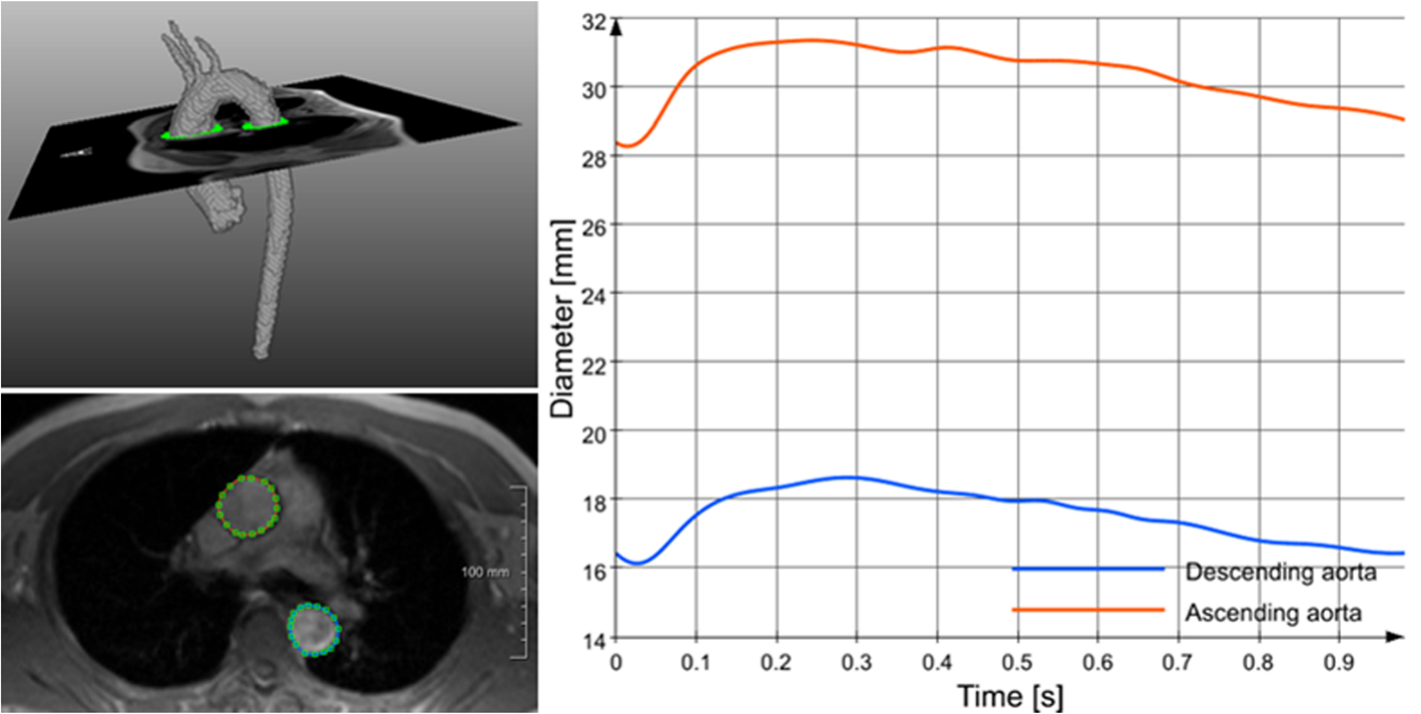
Calculation of local distensibility. **Right**: Positioning of 2D analysis planes of 2D CINE CMR in the ascending and descending aorta. **Middle**: delineation and tracking of the vessel walls in order to follow the diameter change over time. **Right**: subsequent diameter of the AAo and DAo over the cardiac cycle is shown

Calculation of DC started with the manual definition of two regions of interest (ROI) – one for the ascending and one for the descending aorta - in a diastolic image of the 2D CINE CMR data as depicted in Fig. 2. These contours were automatically propagated to all other time points as described previously [19, 20]. In order to transfer the delineation of the ROI to the entire series of CINE images, the vessel motion was estimated using the Morphon approach, a phase-based registration method [20]. The calculated motion was used to propagate the vessel boundaries from the reference frame through the image series. The Morphon approach was applied to compensate for spatial displacements due to cardiac and pulsatile vessel motion between these time frames. This non-rigid registration method estimates the deformation between two frames from the phase difference between quadrature filter responses, which are intensity-invariant and proportional to the spatial change. This calculation is iterated in a scale space to handle both noise and motion in different orders of magnitude. The contours of these reference frames are then propagated through the cardiac cycle using serial deformation fields calculated with the same method. Once the contours are computed, the areas of the ROIs are calculated through voxel summation and then converted to millimeters. Assuming a circular shape, a diameter is estimated from this area at each time point. Figure 2 also depicts the change in the estimated diameter at each time point of the cardiac cycle for the AAo and DAo. After obtaining diameters, DC was calculated as follows:

$$ \mathrm{DC}=\frac{\left(2\times \Delta \mathrm{d}/\mathrm{Dd}\right)}{\Delta \mathrm{P}}{10}^{-3}/\mathrm{kPa} $$

where Δd is the change in diameter of the aorta in systole and end-diastole, Dd the end-diastolic diameter and ΔP the pulse pressure (=systolic – diastolic blood pressure). Blood pressure was measured before and after CMR (see description above) and the average of both systolic and diastolic values were considered for the calculation of DC.

From the total of 126 cases one case had to be removed, as there was no 2D CINE CMR data available. In another case the boundaries of the AAo were not visible for contour delineation. Therefore, calculation of DC was performed in 124 subjects.

### Statistical analysis

Data are presented as mean (±standard deviations) or median (interquartile range) for continuous, absolute, and relative frequencies for categorical variables. Differences between the three calculation algorithms of PWV were analyzed using a Wilcoxon test.

We compared PWV and distensibility coefficient of the AAo, DAo and both using Pearson correlation coefficients. Regression analysis was performed to quantify the effect of age and gender. The residuals from this regression were then correlated with the residuals of regression of other factors on age and gender in order to assess the association beyond the general aging and gender effects. The factors considered were baseline characteristics (men or females, the latter 5 years after menopause, body-mass-index, hypertension, hypercholesterinemia, diabetes, smoking habit, number of risk factors), vital signs (systolic, diastolic and mean blood pressure before and after CMR examination, mean heart rate during CMR), echocardiographic (ejection fraction) and CMR parameter (maximum plaque thickness and diameter of the ascending and descending aorta and the aortic arch). All tests were two-sided with 0.05 as the level of statistical significance. Inter-observer agreement of PWV calculation based on 4D flow CMR data is reported by mean difference and limits of agreement and was displayed using a Bland-Altman plot. Statistical analyses were performed using SPSS Statistics (version 19.0.1, International Business Machines, Armonk, New York, USA) and Stata 14.1.

## Results

### Patients’ characteristics

Baseline characteristics and cardiovascular risk factors are given in Table 1. All participants were Caucasian. Hypertension and hypercholesterolemia were significantly more frequent in older subjects. No other differences between groups in terms of cardiovascular risk factors were found. Only few subjects had diabetes (*n* = 2), prior stroke (*n* = 2), coronary artery disease (*n* = 2), and none of them suffered from peripheral vascular disease.

**Table 1:** Baseline characteristics and cardiovascular risk factors of study participants

Characteristics	*N* = 126
Age, years (±SD)	49.2 (±16.6)
Female, n (%)	64 (50.8)
Hypertension, n (%)	21 (16.7)
Hypercholesterolemia, n (%)	21 (16.7)
Diabetes, n (%)	2 (1.6)
Smoker, n (%)	22 (17.5)
BMI, kg/m^2^ (±SD)	24.8 (±4.1)
Prior stroke, n (%)	2 (1.6)
Coronary heart disease, n (%)	2 (1.6)
Peripheral arterial disease, n (%)	0 (0.0)
Mean systolic BP, mmHg (±SD)	126.6 (±16.3)
Mean diastolic BP, mmHg (±SD)	79.8 (±9.2)
Heart rate, bpm (±SD)	66.3 (±8.2)

*BMI* body mass index, *SD* standard deviation, *BP* blood pressure, *bpm* heart rate

TTE metrics are given in Table 2. TTE values were normal in all but two individuals, with one having a mildly reduced LV systolic function (ejection fraction =45%) and one having an enlarged left atrium (dimension = 49 mm).

**Table 2:** TTE metrics of the study participants

Characteristics	*N* = 126
Left ventricular end-diastolic diameter – mm (±SD)	48.7 (±2.7)
Left ventricular end-systolic diameter – mm (±SD)	31.1 (±5.3)
Systolic ejection fraction – % (±SD)	55.5 (±1.3)
Left atrial diameter – mm (±SD)	34.3 (±5.1)
Aortic root diameter – mm (±SD)	31.9 (±4.6)
Aortic valve regurgitation	
- grade I° – n (%)	5 (4.0)
- grade II° – n (%)	1 (0.8)
Mitral valve insufficiency	
- grade 0 – I° – n (%)	36 (28.6)
- grade I° – n (%)	41 (32.5)
- grade II° – n (%)	2 (1.6)
- grade III° – n (%)	1 (0.8)
Inferior caval vein collapse	
- almost complete – n (%)	7 (5.6)
- no collapse – n (%)	1 (0.8)
Maximum velocity across aortic valve – m/s (±SD)	1.25 (±0.26)
Diameter of the proximal ascending aorta – mm (±SD)	31.0 (±4.0)

Only mean values and pathological values are given. Pulmonary and tricuspid valve metrics are not presented, as they do not influence aortic pulse wave velocity

### Aortic diameters and incidence of aortic atheroma in CMR

**Fig. 3:**
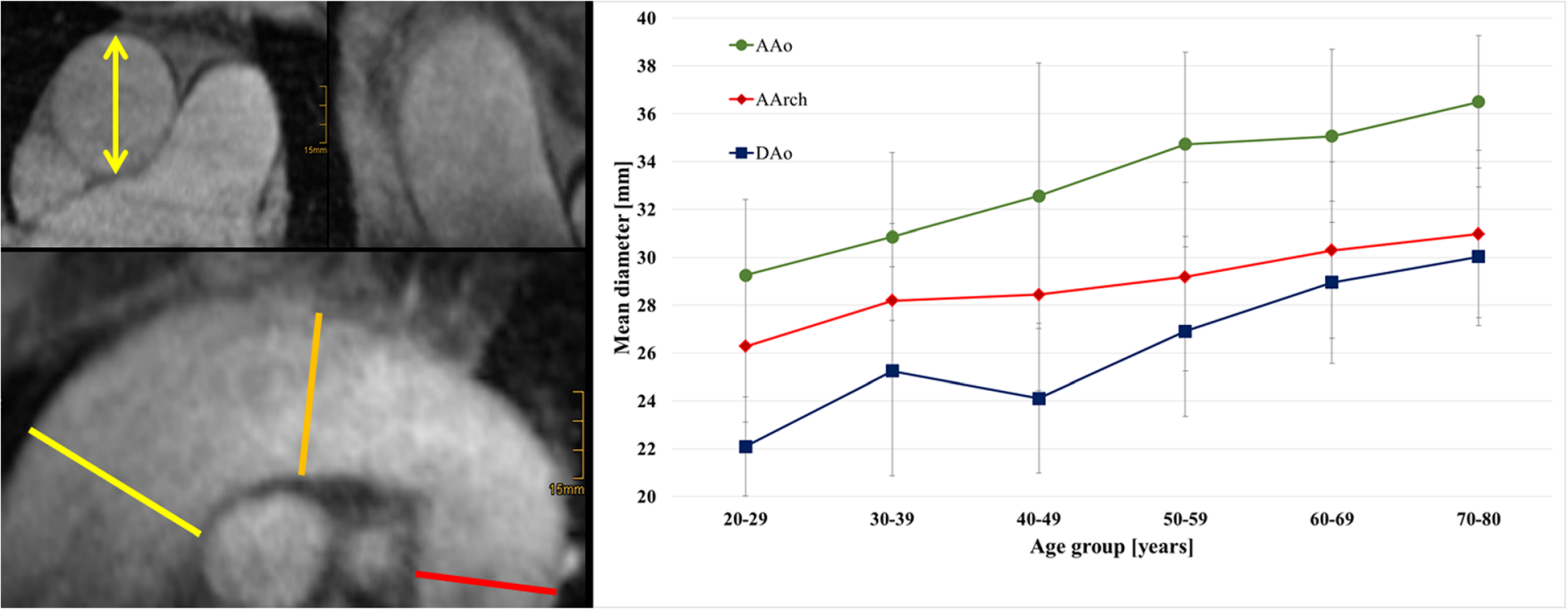
Correlation of aortic diameter with age. **Left**: Maximum aortic diameter was measured based on 3D bright blood T1 weighted CMR using axial slides (yellow arrow) orientated normal to the aorta at the level of the ascending (yellow bar) and descending aorta (red bar) at the level of the pulmonary artery and at the summit of the arch (orange bar) between the vessel outlets. **Right**: Increase of mean diameter of the ascending, arch, and descending aortic diameter with age is shown. Diameters of the ascending aorta and of the aortic arch were not different between adjacent age groups

Maximum diameters of the AAo, aortic arch and DAo are shown in Fig. 3. All three variables increased significantly with age (*p* < 0.0001).

**Fig. 4:**
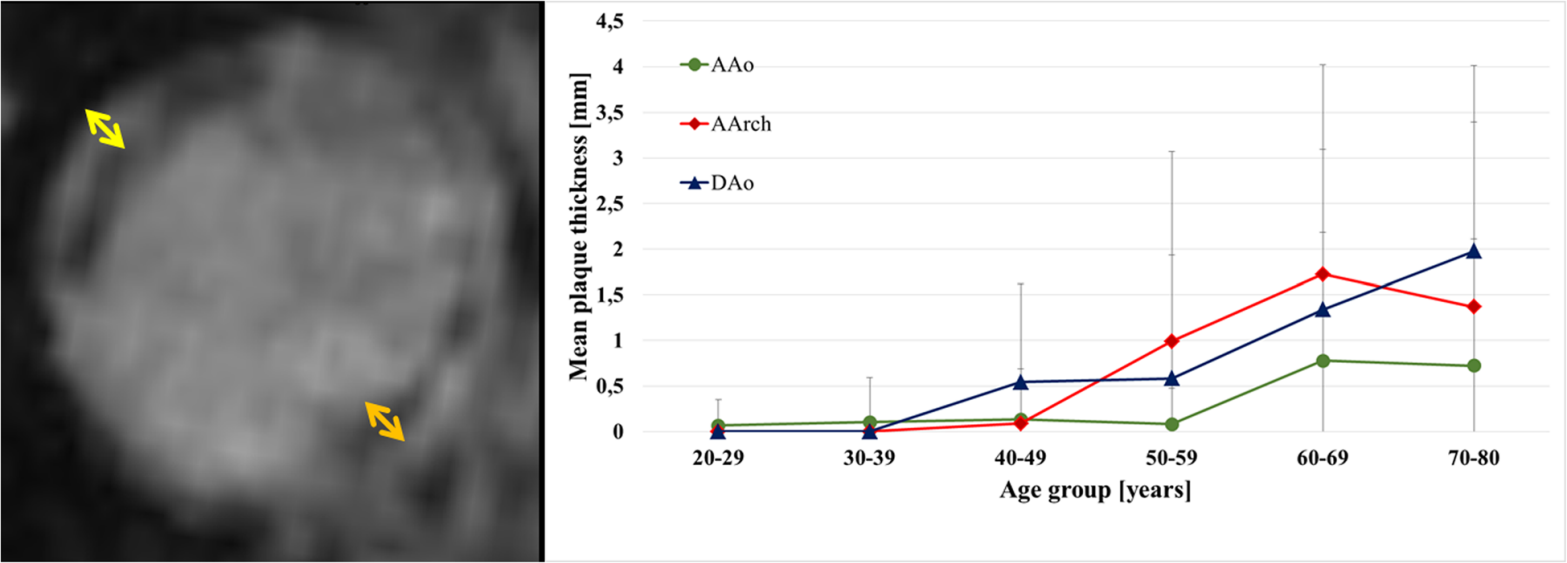
Correlation of aortic wall thickness with age. **Left**: 3D bright blood T1 weighted CMR of the descending aorta (DAo) demonstrates predominantly calcified and ca. 4.5 mm thick plaques (yellow and orange arrows) in a 78-year-old male. **Right**: Increase of maximum plaque thickness in the ascending aorta (AAo), aortic arch (AArch) and DAo with increasing age. Plaques ≥4 mm (i.e. complex plaques) were only found in subjects ≥50 years of age

Forty-one aortic plaques were detected (Fig. 4). Prevalence of plaques < 4 mm was as follows: 1/20 (5%) in 20–29 years of age and 1/23 (4.3%), 4/21 (19.0%), 6/23 (26.1%), 10/21 (47.6%), 4/18 (22.2%) in the subsequent decades. Plaques ≥4 mm were only found from subjects at least 50 years of age and were most frequent in 70–80 year-old patients. Prevalence was 4/23 (17.4%), 4/21 (19.0%) and 7/18 (38.9%) in the decades 50–59, 60–69, and 70–79 years of age.

### Pulse wave velocity calculation

Mean PWV was calculated using the time-to-foot, 50%-rule and cross correlation method. Values were 6.10 ± 1.42 m/s, 6.5 ± 2.01 m/s, and 6.23 ± 1.54 m/s, respectively, and did not differ significantly (*p* = 0.486). Cross correlation showed fewest outliers and was, therefore, considered for all further statistical test.

**Fig. 5:**
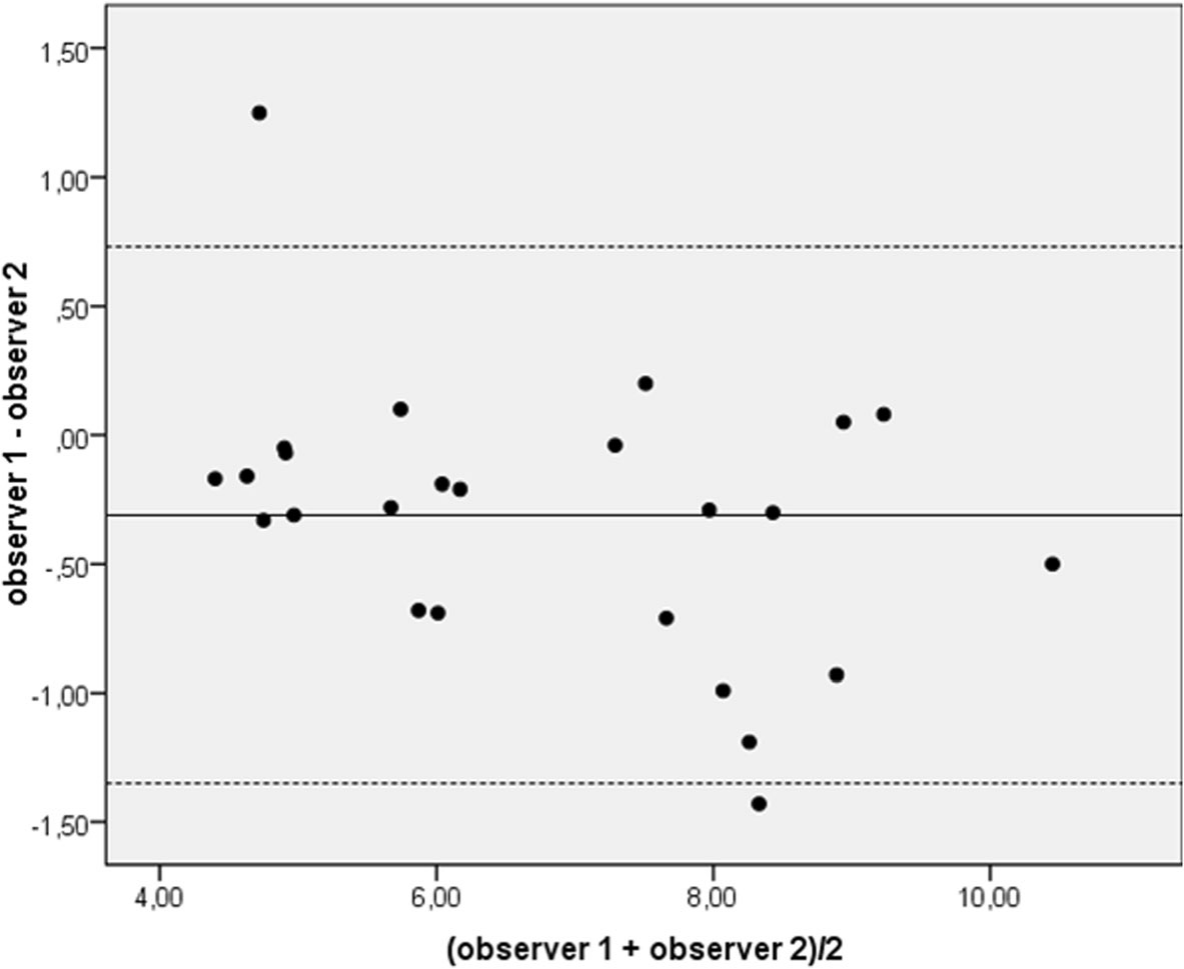
Bland-Altman plot of the inter-observer agreement of PWV in m/s. PWV was calculated based on 4D flow CMR data of 25 subjects of the study cohort. Mean difference (solid line) ± 1.96 x standard deviation (dashed lines) is shown

Inter-observer agreement was based on data sets of 25 randomly selected subjects. This showed a mean difference of − 0.31 (95% CI: [− 0.40,-0.21]) and a standard deviation of 0.53 m/s. Consequently, the limits of agreement (mean difference ± 1.96 x standard deviation) ranged from − 1.35 to 0.73 m/s (see also Bland-Altman plot in Fig. 5). These were moderate when compared to the spread of PWV over the whole age range.

**Fig. 6:**
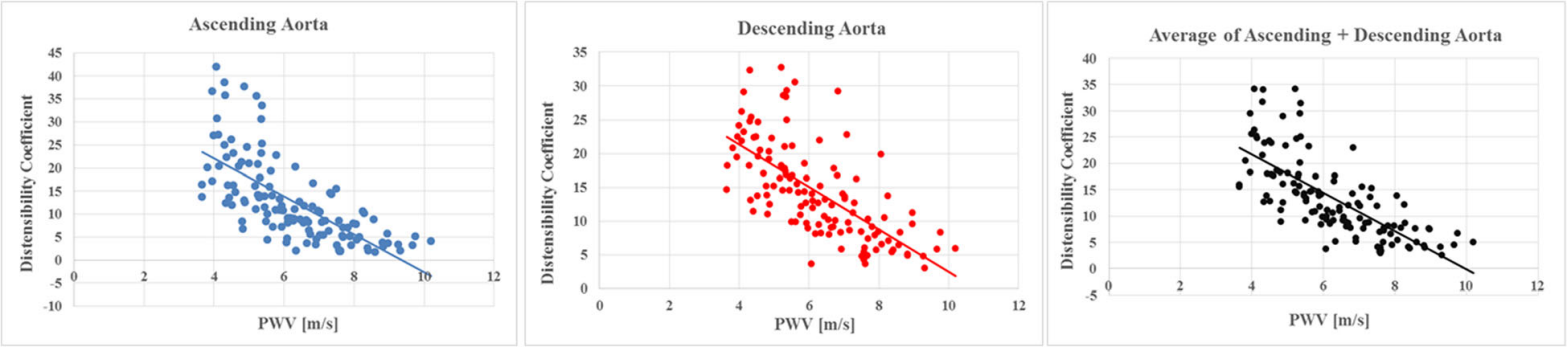
Correlation of pulse wave velocity and distensibility coefficients. PWV was inversely correlated with DC of the ascending and descending aorta and the average of both. PWV = pulse wave velocity, DC = distensibility coefficient

We observed a high negative correlation of PWV with distensibility coefficients for the ascending aorta, the descending aorta, and both (*r* = − 0.69, *r* = − 0.68 and *r* = − 0.73, respectively (*p* < 0.001)) indicating that subjects with an elastic aorta had a low PWV and a high distensibility coefficient (Fig. 6). These negative correlations reflect mainly a joint association with age, as they reduce to *r* = − 0.04, *r* = − 0.09 and *r* = − 0.08, respectively, after adjusting for age and gender.

### Age and gender dependence of pulse wave velocity

**Fig. 7:**
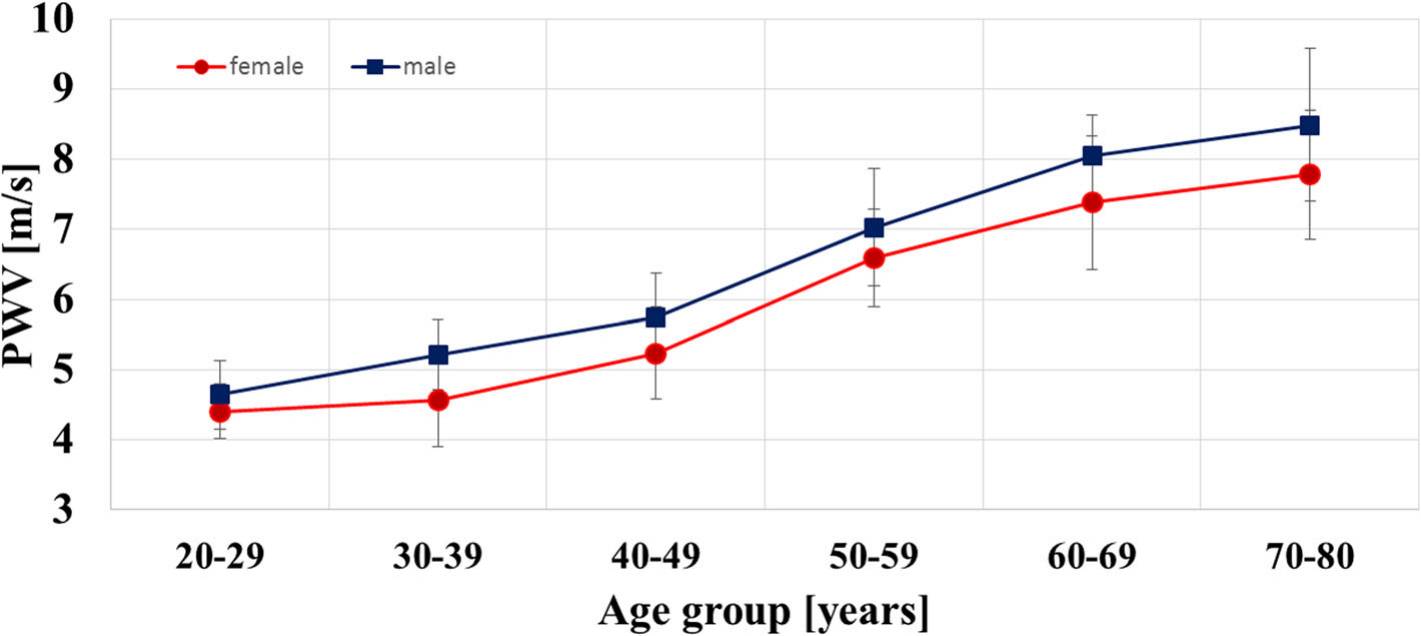
Correlation of aortic pulse wave velocity with age and gender. Mean PWV ± standard deviation is given for each decade for females and males. PWV increased with increasing age and was lower in females compared to males

PWV depended significantly on age (p < 0.001) and gender (*p* < 0.001) (see Table 3 and Fig. 7). PWV increased from 4.93 ± 0.54 m/s in 20–30 to 8.06 ± 1.03 m/s in 70–80 year-old subjects. Values in males were 0.53 fold higher compared to women (95% CI: 0.26–0.80). However, no evidence for a difference of the age effect between men and women could be found (*p* = 0.23). The formula used for age and gender standardization was PWV = 1.51 + 0.080*age (in years) + 0.53*male with a standard deviation of 0.77 m/s.

**Table 3:** Age and gender-dependent values of pulse wave velocity

	20–29 (years)	30–39 (years)	40–49 (years)	50–59 (years)	60–69 (years)	70–79 (years)
Females	*N* = 10	*N* = 9	*N* = 11	*N* = 13	*N *= 12	*N* = 9
PWV (m/s)	4.41 ± 0.39	4.57 ± 0.67	5.24 ± 0.66	6.59 ± 0.70	7.38 ± 0.95	7.78 ± 0.93
Males	N = 10	*N* = 14	*N* = 10	N = 10	N = 9	*N* = 9
PWV (m/s)	4.64 ± 0.49	5.22 ± 0.50	5.57 ± 0.63	7.03 ± 0.84	8.05 ± 0.57	8.49 ± 1,09

*PWV* pulse wave velocity

± indicates standard deviation

### Associations beyond age and gender

Increased blood pressure (systolic *r* = 0.35/0.41, diastolic *r* = 0.32/0.35, before/after CMR examination and mean arterial pressure *r* = 0.38; *p* < 0.0001) correlated with PWV after adjustment for age. Finally, there was positive correlation with the diameter of the AAo (*r* = 0.20, *p* = 0.026), aortic arch (*r* = 0.24, *p* = 0.009), and of the DAo (*r* = 0.26, *p* = 0.004) with PWV after adjustment for age (Table 4).

**Table 4:** Correlation of baseline and CMR parameter with pulse wave velocity after adjustment for age and gender

Characteristic	Correlation Coefficient (*r*)	*P* value
Average systolic blood pressure (before/after MRI)	0.36	0.0000
Average diastolic blood pressure (before/after MRI)	0.33	0.0001
Mean arterial blood pressure (before/after MRI)	0.37	0.0000
Average heart rate (during MRI)	0.14	0.121
Body mass index	0.12	0.184
Diameter aortic root in echocardiography	0.08	0.396
Maximum plaque thickness aorta in MRI	0.04	0.708
Diameter ascending aorta in MRI	0.20	0.026
Diameter aortic arch in MRI	0.24	0.009
Diameter descending aorta in MRI	0.26	0.004

## Discussion

We performed a prospective population-based study and selectively calculated aortic PWV based on a novel method using 4D flow CMR and dedicated software. To the best of our knowledge, this is the first study using this approach while providing age- and gender related normative values of aortic stiffness. In addition to cardiovascular risk factors, we compared wall thickness and diameter throughout the thoracic aorta using 3D CMR, which is superior to TEE for the thorough detection of aortic atherosclerosis [21], with PWV derived from 4D flow CMR measurements.

The advantage of 4D flow CMR is the ability to selectively study the aorta and to include complex geometries such as elongations that are not assessable using multiple 2D CMR analysis planes. Previous 2D CMR studies used a temporal resolution of ~ 10 ms [9]. The chosen 20 ms temporal resolution in our study was lower but this was outweighed by averaging PWV based on 50–60 instead of only 2–5 analysis planes in previous 2D approaches. The high reliability and good reproducibility of 4D flow CMR at even lower temporal resolution (40 ms) was previously shown [10–12]. In addition, inter-observer agreement of PWV calculation in 25 randomly selected subjects of our population study was moderate underlining accuracy of this technique. In the present study, we were able to calculate PWV based on automatically generated analysis planes along the automatically positioned centerline with predefined inter-plane distance. In terms of distance calculation, this procedure is superior to carotid-femoral tonometry relying on indirect measurement of the interspace between two measurement points. Findings were compared with the distensibility coefficient as an alternative method for measuring local aortic compliance based on 2D analysis planes. We found a high inverse correlation of both techniques, i.e. PWV increases and DC decreases with increasing age. This close relationship of PWV with another biomarker of vessel compliance thus indirectly demonstrates the robust assessment of aortic compliance based on 4D flow CMR.

We included a large cohort of healthy subjects between 20 and 80 years and performed both 3D CMR and routine TTE for study reasons. Therefore, we are able to provide representative data for aortic stiffness, diameter, and plaques in a general population. Comparable to other population-based studies our participants were relatively healthy, which is evident from the low incidence of cardiovascular risk factors, normal TTE in almost all subjects and normal diameters of the aorta with only few plaques ≥4 mm. This is due to the higher motivation of health-conscious people to participate in such a study compared for example to patients with advanced cardiovascular disease due to modifiable risk factors.

PWV in our population-based study ranged from 5 m/s in the youngest (20–29 years) to 8 m/s in the oldest (70–79 years) when using 4D flow CMR. Hickson et al. [22] conducted a study in 162 subjects (mean age 18–77 years) free from cardiovascular disease and medication and measured PWV using 2D CMR. PWV of the arch and descending aorta was ca. 4 m/s in 20–30 year-olds and 6-7 m/s in 70–80 year-olds. Carotid-femoral pulse wave velocity calculation based on tonometry was ca. 5.5 and 9 m/s in these age groups and thus higher compared to CMR. However, it is well known that the measurement principle of tonometry yields (nominal) values for PWV, which are systematically higher than the true propagation velocity of aortic PWV due to the inclusion of the stiffer carotid, iliac and femoral arteries. PWV values of our population including relatively few subjects with cardiovascular risk factors or advanced aortic atherosclerosis are similar and thus highly plausible.

The population-based study by Redheuil et al. [6] included 111 asymptomatic subjects free of acute or chronic disease and measured PWV using two single 2D MR analysis planes in the ascending and descending aorta. Subjects in the third decade of their lives had a PWV of ~ 3.5 m/s increasing to 11 m/s for those in their seventh decade. 4D flow CMR based PWV calculations in 86 acute stroke patients revealed a mean velocity of 5.8 m/s compared to 3.8 m/s in 17 healthy and younger subjects [10]. Dyverfeldt et al. used 4D flow CMR in eight healthy subjects and patients and reported similar values [23].

Finally, Vlachopoulos et al. [7] pooled heterogeneous data from > 10.000 subjects of general populations or community-based adults and of > 5.500 patients with cardiovascular diseases. Aortic PWV was determined by Doppler flow or carotid-femoral tonometry. In the general populations or community-based old adults included in this meta-analysis, mean PWV was 9.5 and 11.3 m/s in those with a mean age of 52 or 55 years, respectively, and increased to 9.0 and 13 m/s in subjects with a mean age of 72 or 74 years, respectively. Patients with manifest cardiovascular disease showed PWV of 11 m/s at a mean age of ca. 50 years, which was essentially higher compared to values obtained in our study. The overall higher PWVs are most likely due to differences in cohorts, severity of atherosclerotic diseases, and measurement methods.

Our statistical analysis indicated that age, especially, was responsible for an increase of PWV, which is in line with previous studies [5–7, 22–25]. One main hypothesis for this observation is related to fatigue fractures of elastin fibers [19], which are particularly concentrated in the proximal part of the thoracic aorta (i.e. ascending aorta and arch) serving as a Windkessel under physiological conditions. Other classical risk factors such as hypertension had only minor influence on PWV. In our cohort, increased aortic diameter correlated with an increase of PWV after adjustment for age and gender. This can be explained by the fact that atherosclerosis in such patients led to outward remodeling of the aorta with concomitant stiffening. By contrast, maximum aortic plaque thickness in 3D CMR showed only low correlation with PWV (*r* = 0.04) and was not an independent predictor of increased stiffness although it is another robust parameter of aortic atherosclerosis. This is most probably due to the limited cohort size and the number of subjects with advanced atherosclerosis, which was too small to show such positive effects. Interestingly, mean PWV was lower in females than in males and this could be due to hormonal influence on aortic wall structure. For a more detailed analysis, however, our cohort was not large enough. Nevertheless, such gender differences should be further investigated and especially considered when assessing aortic stiffness using our reference values in future patients. Interestingly, smoking habits and obesity were no predictors of increased PWV. However, only 17.5% of our participants were smokers and only 10% of the subjects were obese. Thus, our cohort did not provide optimal conditions to study such effects and larger cohorts including more patients with severe cardiovascular risk factors including obesity are needed to investigate such influence via the proposed CMR methodology.

A limitation of our study is the lack of a comparison with aortic catheterization, carotid-femoral tonometry and 2D PC CMR. This would underline measurement accuracy of our approach and determine differences in PWV calculations that are directly related to the technique applied. In particular, the comparison with invasive catheterization as the reference method would be highly valuable to determine or rule out potential measurement errors of 4D flow CMR and could be executed in patients undergoing routine diagnostic cardiac catheterization. Due to the concordance of our PWV findings with previous studies in terms of age and cardiovascular risk factors we believe that our age- and gender-related normal data are plausible, robust, and accurate. Moreover, values showed a high correlation with DC, another established marker of local compliance, of the ascending and descending aorta. PWV depends on the composition of the examined population and may differ to a certain extent at different sites. Therefore, other research groups should reproduce our normal values in order to increase the amount of available reference values for future comparison in patients with aortic diseases. PWV calculation may also depend on the hardware and software platform used. Thus, a validation and comparison with other CMR systems and software approaches should be performed in the future. In addition, the presented method has the potential to non-invasively calculate even local PWV in the ascending and descending aorta or in the aortic arch and thus allow further insight into pathophysiology of aortic atherosclerosis. This option is currently only provided by invasive aortic catheterization. Due to the reduction of total length of the aortic segments, however, a further increase of temporal resolution below 10 ms and thus a longer measurement time of 4D flow CMR would be necessary.

## Conclusions

We have shown that 4D flow CMR can be used to directly and selectively assess aortic PWV avoiding the inherent systematic errors of carotid-femoral tonometry. The image analysis strategy used here provides values of PWV calculated by different algorithms within minutes and is, therefore, suited for analysis in research or in the clinical setting. PWV increases with age and in association with hypertension and increased aortic diameter. Knowledge of such factors and availability of normal data is very helpful to identify subjects with increased risk for the development of cardiovascular disease and to optimally monitor treatment effects in patients in time. The additional advantage of 4D flow CMR is the possibility to calculate further hemodynamic parameters based on the acquired data sets such as blood flow velocity and volume, wall shear stress, and embolization pathways [26]. In addition, it allows direct correlation with 3D CMR for the assessment of vessel diameter and thickening as demonstrated here. Accordingly, it can be applied for a comprehensive investigation of the individual aorta and risk of atherosclerosis development and progression.

## Abbreviations

4D: Four-dimensional
AAo: Ascending Aorta
CINE-MRI: Cinematographic magnetic resonance imaging
DAo: Descending Aorta
ECG: Electrocardiogram
EF: Ejection Fraction
GRAPPA: GeneRalized Autocalibrating Partial Parallel Acquisition
LV: Left Ventricle/left Ventricular
PEAK: Parallel MRI with Extended and Averaged GRAPPA Kernels
PWV: Pulse wave velocity
RV: Right Ventricle/right Ventricular
TTE: Transthoracic echocardiography
TTF: Time-to-foot
Xcor: Cross-correlation

## References

[1] Sethi S, Rivera O, Oliveros R, Chilton R (2014). Aortic stiffness: pathophysiology, clinical implications, and approach to treatment. Integr Blood Press Control.

[2] Smulyan H, Mookherjee S, Safar ME (2016). The two faces of hypertension: role of aortic stiffness. J Am Soc Hypertens.

[3] Townsend RR, Wilkinson IB, Schiffrin EL (2015). American Heart Association Council on hypertension. Recommendations for improving and standardizing vascular research on arterial stiffness. A scientific statement from the American Heart Association. Hypertension.

[4] Mancia G, Fagard R, Narkiewicz K (2013). ESH/ESC guidelines for the management of arterial hypertension. The task force for the management of arterial hypertension of the European Society of Hypertension (ESH) and of the European Society of Cardiology (ESC). Eur Heart J 2013.

[5] Ben-Shlomo Y, Spears M, Boustred C (2014). Aortic pulse wave velocity improves cardiovascular event prediction: an individual participant meta-analysis of prospective observational data from 17,635 subjects. J Am Coll Cardiol.

[6] Redheuil A, Wu CO, Kachenoura N (2014). Proximal aortic distensibility is an independent predictor of all-cause mortality and incident CV events: the MESA study. J Am Coll Cardiol.

[7] Vlachopoulos C, Aznaouridis K, Stefanadis C (2010). Prediction of cardiovascular events and all-cause mortality with arterial stiffness: a systematic review and meta-analysis. J Am Coll Cardiol.

[8] Voges I, Jerosch-Herold M, Hedderich J (2012). Normal values of aortic dimensions, distensibility, and pulse wave velocity in children and young adults: a cross-sectional study. J Cardiovasc Magn Reson.

[9] Wentland AL, Grist TM, Wieben O (2014). Review of MRI-based measurements of pulse wave velocity: a biomarker of arterial stiffness. Cardiovasc Diagn Ther.

[10] Markl M, Wallis W, Brendecke S (2010). Estimation of global aortic pulse wave velocity by flow-sensitive 4D MRI. Magn Reson Med.

[11] Markl M, Wallis W, Strecker C (2012). Analysis of pulse wave velocity in the thoracic aorta by flow-sensitive four-dimensional MRI: reproducibility and correlation with characteristics in patients with aortic atherosclerosis. J Magn Reson Imaging.

[12] Wentland AL, Wieben O, François CJ (2013). Aortic pulse wave velocity measurements with undersampled 4D flow-sensitive MRI: comparison with 2D and algorithm determination. J Magn Reson Imaging.

[13] Lang RM, Bierig M, Devereux RB (2005). Recommendations for chamber quantification: a report from the American Society of Echocardiography’s guidelines and standards committee and the chamber quantification writing group, developed in conjunction with the European Association of Echocardiograph. J Am Soc Echocardiogr.

[14] Markl M, Harloff A, Bley TA (2007). Time-resolved 3D MR velocity mapping at 3T: improved navigator-gated assessment of vascular anatomy and blood flow. J Magn Reson Imaging.

[15] Tunick PA, Kronzon I (2000). Atheromas of the thoracic aorta: clinical and therapeutic update. J Am Coll Cardiol.

[16] Wehrum T, Kams M, Schroeder L (2014). Accelerated analysis of three-dimensional blood flow of the thoracic aorta in stroke patients. Int J Cardiovasc Imaging.

[17] Drexl J, Mirzaee H, Harloff A (2013). A software tool for the computation of arterial pulse wave velocity from flow-sensitive 4D MRI data. Comput Cardiol.

[18] Harloff A, Strecker C, Reinhard M (2006). Combined measurement of carotid stiffness and intima-media thickness improves prediction of complex aortic plaques in patients with ischemic stroke. Stroke.

[19] Hüllebrand M, Hennemuth A, Messroghli D, Kühne T (2014). An OsiriX plugin for integrated cardiac image processing research. Proc SPIE Med Imaging.

[20] Tautz L, Hennemuth A, Peitgen HO, Camara O, Mansi T, Pop M, Rhode K, Sermesant M, Young A (2011). Motion analysis with quadrature filter based registration of tagged MRI sequences. Statistical atlases and computational models of the heart. Imaging and modelling challenges.

[21] Harloff A, Brendecke SM, Simon J (2012). 3D MRI provides improved visualization and detection of aortic arch plaques compared to transesophageal echocardiography. J Magn Reson Imaging.

[22] Hickson SS, Butlin M, Graves M (2010). The relationship of age with regional aortic stiffness and diameter. JACC Cardiovasc Imaging.

[23] Dyverfeldt P, Ebbers T, Länne T (2014). Pulse wave velocity with 4D flow MRI: systematic differences and age-related regional vascular stiffness. Magn Reson Imaging.

[24] Ohyama Y, Teixido-Tura G, Ambale-Venkatesh B (2016). Ten-year longitudinal change in aortic stiffness assessed by cardiac MRI in the second half of the human lifespan: the multi-ethnic study of atherosclerosis. Eur Heart J Cardiovasc Imaging.

[25] Nethononda RM, Lewandowski AJ, Stewart R (2015). Gender specific patterns of age-related decline in aortic stiffness: a cardiovascular magnetic resonance study including normal ranges. J Cardiovasc Magn Reson.

[26] Dyverfeldt P, Bissell M, Barker AJ (2015). 4D flow cardiovascular magnetic resonance consensus statement. J Cardiovasc Magn Reson.

